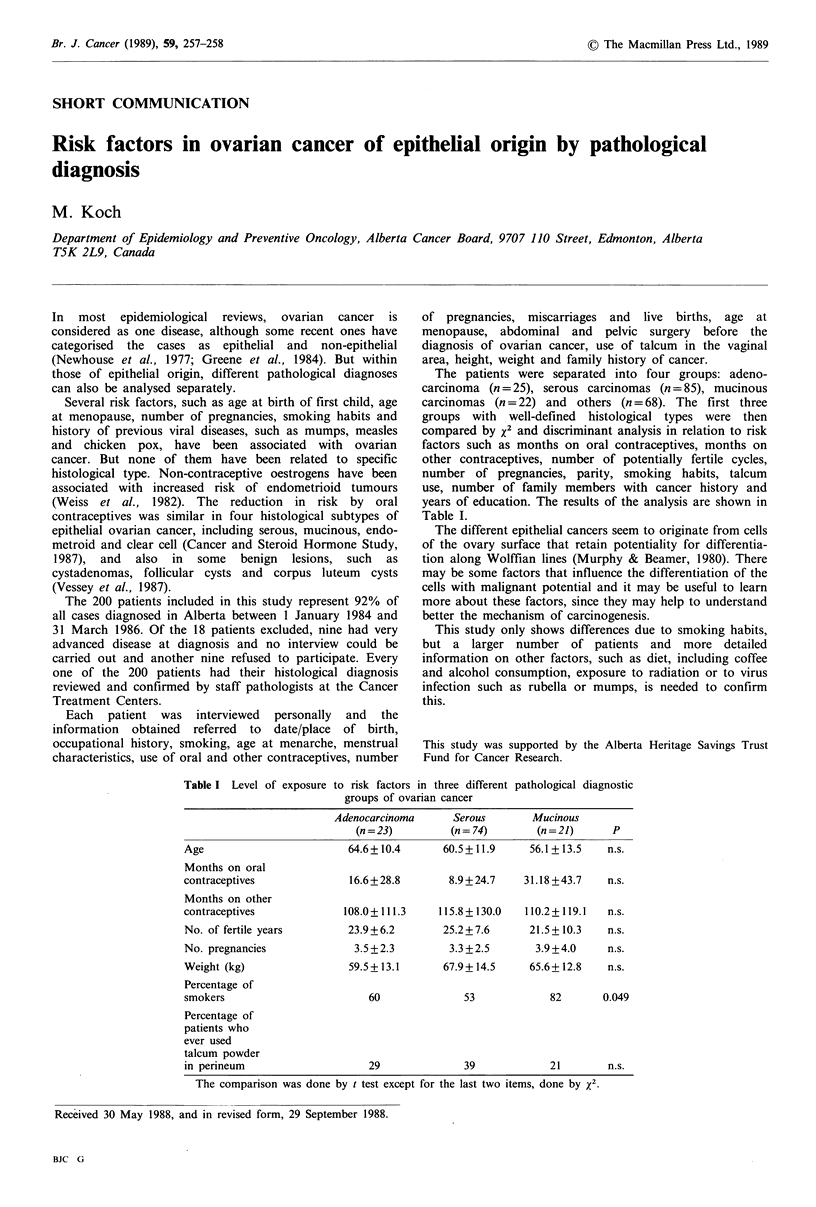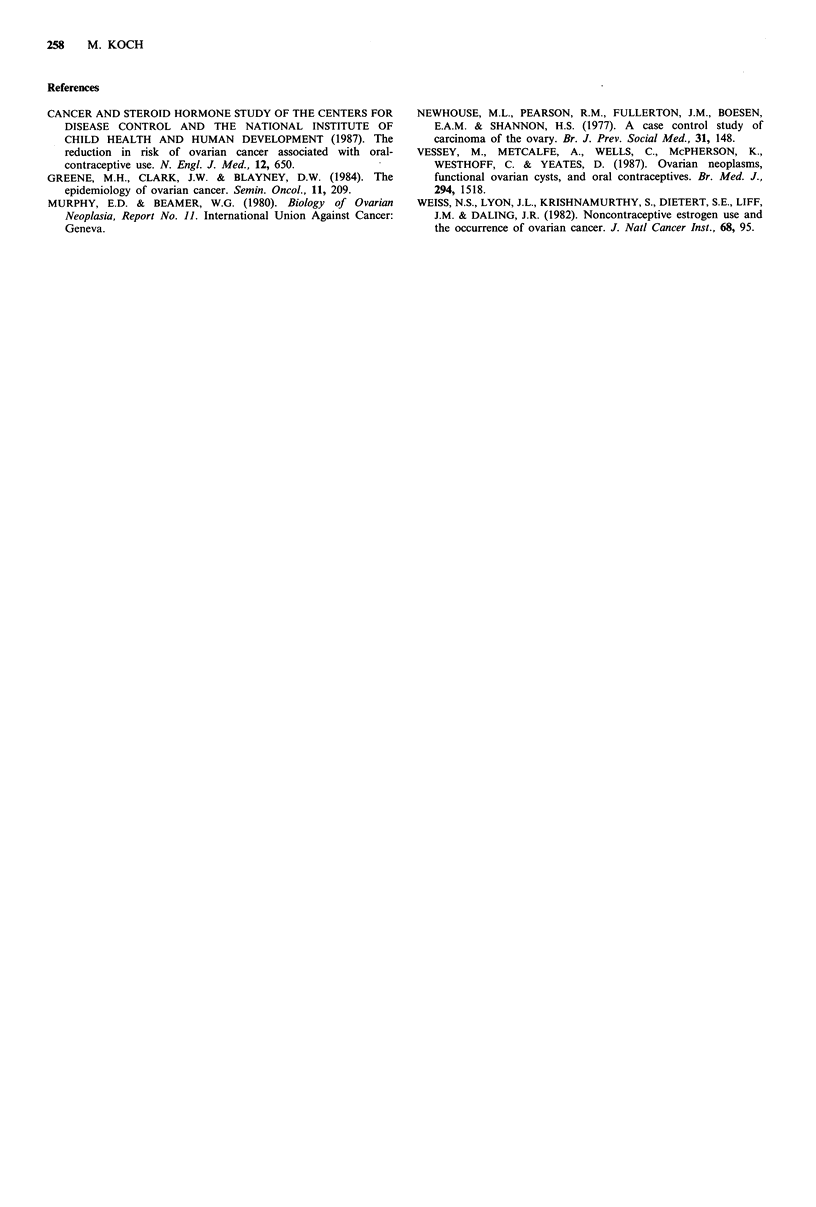# Risk factors in ovarian cancer of epithelial origin by pathological diagnosis.

**DOI:** 10.1038/bjc.1989.53

**Published:** 1989-02

**Authors:** M. Koch

**Affiliations:** Department of Epidemiology and Preventive Oncology, Alberta Cancer Board, Edmonton, Canada.


Br. J. Cancer (1989), 59, 257–258 © The Macmillan Press Ltd., 1989

SHORT COMMUNICATION

### Risk factors in ovarian cancer of epithelial origin by pathological diagnosis

M. Koch

*Department of Epidemiology and Preventive Oncology, Alberta Cancer Board, 9707 110 Street, Edmonton, Alberta T5K 2L9, Canada*

In most epidemiological reviews, ovarian cancer is considered as one disease, although some recent ones have categorised the cases as epithelial and non-epithelial (Newhouse *et al.*, 1977; Greene *et al.*, 1984). But within those of epithelial origin, different pathological diagnoses can also be analysed separately.

Several risk factors, such as age at birth of first child, age at menopause, number of pregnancies, smoking habits and history of previous viral diseases, such as mumps, measles and chicken pox, have been associated with ovarian cancer. But none of them have been related to specific histological type. Non-contraceptive oestrogens have been associated with increased risk of endometrioid tumours (Weiss *et al.*, 1982). The reduction in risk by oral contraceptives was similar in four histological subtypes of epithelial ovarian cancer, including serous, mucinous, endometroid and clear cell (Cancer and Steroid Hormone Study, 1987), and also in some benign lesions, such as cystadenomas, follicular cysts and corpus luteum cysts (Vessey *et al.*, 1987).

The 200 patients included in this study represent 92% of all cases diagnosed in Alberta between 1 January 1984 and 31 March 1986. Of the 18 patients excluded, nine had very advanced disease at diagnosis and no interview could be carried out and another nine refused to participate. Every one of the 200 patients had their histological diagnosis reviewed and confirmed by staff pathologists at the Cancer Treatment Centers.

Each patient was interviewed personally and the information obtained referred to date/place of birth, occupational history, smoking, age at menarche, menstrual characteristics, use of oral and other contraceptives, number of pregnancies, miscarriages and live births, age at menopause, abdominal and pelvic surgery before the diagnosis of ovarian cancer, use of talcum in the vaginal area, height, weight and family history of cancer.

The patients were separated into four groups: adenocarcinoma ($n=25$), serous carcinomas ($n=85$), mucinous carcinomas ($n=22$) and others ($n=68$). The first three groups with well-defined histological types were then compared by $\chi^2$ and discriminant analysis in relation to risk factors such as months on oral contraceptives, months on other contraceptives, number of potentially fertile cycles, number of pregnancies, parity, smoking habits, talcum use, number of family members with cancer history and years of education. The results of the analysis are shown in Table I.

The different epithelial cancers seem to originate from cells of the ovary surface that retain potentiality for differentiation along Wolffian lines (Murphy & Beamer, 1980). There may be some factors that influence the differentiation of the cells with malignant potential and it may be useful to learn more about these factors, since they may help to understand better the mechanism of carcinogenesis.

This study only shows differences due to smoking habits, but a larger number of patients and more detailed information on other factors, such as diet, including coffee and alcohol consumption, exposure to radiation or to virus infection such as rubella or mumps, is needed to confirm this.

This study was supported by the Alberta Heritage Savings Trust Fund for Cancer Research.

**Table I** Level of exposure to risk factors in three different pathological diagnostic groups of ovarian cancer

| | *Adenocarcinoma* ($n=23$) | *Serous* ($n=74$) | *Mucinous* ($n=21$) | *P* |
|---|---|---|---|---|
| Age | 64.6±10.4 | 60.5±11.9 | 56.1±13.5 | n.s. |
| Months on oral contraceptives | 16.6±28.8 | 8.9±24.7 | 31.18±43.7 | n.s. |
| Months on other contraceptives | 108.0±111.3 | 115.8±130.0 | 110.2±119.1 | n.s. |
| No. of fertile years | 23.9±6.2 | 25.2±7.6 | 21.5±10.3 | n.s. |
| No. pregnancies | 3.5±2.3 | 3.3±2.5 | 3.9±4.0 | n.s. |
| Weight (kg) | 59.5±13.1 | 67.9±14.5 | 65.6±12.8 | n.s. |
| Percentage of smokers | 60 | 53 | 82 | 0.049 |
| Percentage of patients who ever used talcum powder in perineum | 29 | 39 | 21 | n.s. |

The comparison was done by $t$ test except for the last two items, done by $\chi^2$.

Received 30 May 1988, and in revised form, 29 September 1988.

### References

CANCER AND STEROID HORMONE STUDY OF THE CENTERS FOR DISEASE CONTROL AND THE NATIONAL INSTITUTE OF CHILD HEALTH AND HUMAN DEVELOPMENT (1987). The reduction in risk of ovarian cancer associated with oral-contraceptive use. *N. Engl. J. Med.,* **12,** 650.

GREENE, M.H., CLARK, J.W. & BLAYNEY, D.W. (1984). The epidemiology of ovarian cancer. *Semin. Oncol.,* **11,** 209.

MURPHY, E.D. & BEAMER, W.G. (1980). *Biology of Ovarian Neoplasia, Report No. 11.* International Union Against Cancer: Geneva.

NEWHOUSE, M.L., PEARSON, R.M., FULLERTON, J.M., BOESEN, E.A.M. & SHANNON, H.S. (1977). A case control study of carcinoma of the ovary. *Br. J. Prev. Social Med.,* **31,** 148.

VESSEY, M., METCALFE, A., WELLS, C., McPHERSON, K., WESTHOFF, C. & YEATES, D. (1987). Ovarian neoplasms, functional ovarian cysts, and oral contraceptives. *Br. Med. J.,* **294,** 1518.

WEISS, N.S., LYON, J.L., KRISHNAMURTHY, S., DIETERT, S.E., LIFF, J.M. & DALING, J.R. (1982). Noncontraceptive estrogen use and the occurrence of ovarian cancer. *J. Natl Cancer Inst.,* **68,** 95.